# Cellular Therapy for Children with Central Nervous System Tumors: Mining and Mapping the Correlative Data

**DOI:** 10.1007/s11912-023-01423-3

**Published:** 2023-05-09

**Authors:** Erin E. Crotty, Ashley L. Wilson, Tom Davidson, Sophia Tahiri, Juliane Gust, Andrea M. Griesinger, Sujatha Venkataraman, Julie R. Park, Sabine Mueller, Brian R. Rood, Eugene I. Hwang, Leo D. Wang, Nicholas A. Vitanza

**Affiliations:** 1 Ben Towne Center for Childhood Cancer Research, Seattle Children’s Research Institute, M/S JMB-8, 1900 9thAvenue, Seattle, WA 98101 USA; 2 Department of Pediatrics, Seattle Children’s Hospital, University of Washington, Seattle, WA USA; 3 Seattle Children’s Therapeutics, Seattle, WA USA; 4 Cancer and Blood Disease Institute, Keck School of Medicine, Children’s Hospital Los Angeles, University of Southern California, Los Angeles, CA USA; 5 Division of Pediatric Neurology, Department of Neurology, University of Washington, Seattle, WA USA; 6 Center for Integrative Brain Research, Seattle Children’s Research Institute, Seattle, WA USA; 7 Morgan Adams Foundation Pediatric Brain Tumor Research Program, Children’s Hospital Colorado, Aurora, CO USA; 8 Department of Pediatrics, University of Colorado Anschutz Medical Campus, Aurora, CO USA; 9 Department of Neurology, Neurosurgery, and Pediatrics, University of California San Francisco, San Francisco, CA USA; 10 Center for Cancer and Blood Disorders, Children’s National Hospital, Washington, DC USA; 11 Departments of Pediatrics and ImmunoOncology, City of Hope, Duarte, CA USA; 12 Department of Laboratory Medicine and Pathology, University of Washington, Seattle, WA USA

**Keywords:** Pediatric CNS tumors, Adoptive cellular therapy, Immunotherapy, Biomarkers, Correlative studies

## Abstract

**Purpose of Review:**

Correlative studies should leverage clinical trial frameworks to conduct biospecimen analyses that provide insight into the bioactivity of the intervention and facilitate iteration toward future trials that further improve patient outcomes. In pediatric cellular immunotherapy trials, correlative studies enable deeper understanding of T cell mobilization, durability of immune activation, patterns of toxicity, and early detection of treatment response. Here, we review the correlative science in adoptive cell therapy (ACT) for childhood central nervous system (CNS) tumors, with a focus on existing chimeric antigen receptor (CAR) and T cell receptor (TCR)-expressing T cell therapies.

**Recent Findings:**

We highlight long-standing and more recently understood challenges for effective alignment of correlative data and offer practical considerations for current and future approaches to multi-omic analysis of serial tumor, serum, and cerebrospinal fluid (CSF) biospecimens. We highlight the preliminary success in collecting serial cytokine and proteomics from patients with CNS tumors on ACT clinical trials.

## Introduction

In the USA, there are currently more than 30,000 children living with central nervous system (CNS) tumors, making it the most prevalent solid tumor and leading cause of cancer-related death in children [1, 2]. Patient outcomes for malignant CNS tumors remain poor, so - after decades exhausting the benefit of resection, radiation, chemotherapy, and molecularly targeted agents - new therapeutic avenues are desperately needed. Adoptive cellular therapy (ACT), designed to harness the tumor-killing potential of the immune system, is a promising approach with the potential to improve survival in CNS tumor patients while preserving quality of life [3–7]. The field of ACT for CNS tumors is in its infancy, and fundamental questions about the biologic activity of cellular therapies against these tumors remain. Ongoing clinical trials aim to test strategies for timing, dose, and route of ACT delivery, while embedded correlative studies provide the opportunity to gain crucial insights into effector cell expansion and persistence in the CNS, tumor antigen escape, local immunity in the neuroaxis, and the predictive value of biomarker monitoring.

The challenges and perceived opportunities for ACT against CNS tumors have been well documented [3–5]. Hematologic malignancies were the first pediatric cancers to show dramatic clinical responses to cellular therapy, specifically CD19-directed CAR T cells, which induced durable responses in children and adults [8, 9]. An early obstacle in bringing ACT to pediatric CNS tumors has been target selection in the setting of tumor heterogeneity, with initially selected targets focused on the surface antigens B7-H3, EGFRvIII, GD2, HER2, and IL-13Ra2 [10, 11•, 12•, 13•]. Preclinical studies, while often limited by immunocompromised models, have been performed against many CNS tumors, including glioblastoma (GBM) [14•, 15, 16], diffuse midline glioma (DMG) [12•, 17], and atypical teratoid rhabdoid tumor (ATRT) [18•], with encouraging preclinical efficacy leading to phase 1 clinical trials for children and young adults investigating CAR T cells specific to those antigens. While these trials are ongoing, recently published preliminary reports have suggested feasibility, tolerability, safety, and local immune activation in patients using intracranial HER2 [19••], GD2 [20••], and B7-H3-directed CAR T cells [21••]. In addition to CAR T cell therapy, clinical investigations of TCR-expressing T cells targeting H3.3K27M (NCT05478837) and multi-antigen-associated cytotoxic T lymphocytes (TAA-T) targeting WT1, PRAME, and survivin (NCT03652545) [22] are currently being explored.

Due to the variety of antigens targeted, along with differences in eligibility criteria in pediatric cellular therapy trials, identifying common features in immune responses across trials likely requires tight alignment of correlative data analyses. Correlative studies can be broadly defined as investigations that utilize serially collected biospecimens (e.g., cerebrospinal fluid (CSF) or blood) or clinical data (e.g., neuroimaging or clinical assessments) to uncover an underlying bioactivity and effectiveness of the trial intervention. Challenges in collecting shareable data have inspired the creation of cooperative working groups to develop guidelines for upstream sample operations and to determine the best metrics to measure tumor evolution, on-target and off-target toxicity, and disease response. The Consortium for Pediatric Cellular Immunotherapy (CPCI) is a network of collaborating institutions delivering cutting-edge cellular and gene therapies for children through multicenter clinical trials. In addition to coordinating clinical practice, the consortium fosters a real-time review of the current state of the literature and lessons learned from ongoing trials to better inform the next generation of ACT approaches [23••]. Here, CPCI members and research partners review our current understanding of the best practices for correlative analyses on ACT trials for children with brain and spinal cord tumors.

## Radiographic Endpoints

All pediatric CNS ACT trials incorporate standard neuroimaging correlates; however, there is an acknowledged challenge in interpreting these studies in the setting of acute immunotherapeutic delivery. This is compounded in cross-trial comparisons due to the inherent variability in timing, radiographic sequences, and neuroaxis coverage. Traditionally, radiographic response criteria for CNS tumors have been based on the historical experience evaluating cytotoxic therapies, leading to unreliable response data for immunotherapeutic approaches [24, 25]. Specifically, differentiating treatment-related inflammation (i.e., pseudoprogression) from tumor progression or tumor response remains an ongoing challenge. Absent correctives, it can lead to therapeutic mismanagement of patients and erroneous interpretations of early phase clinical trial results. As the field of neuro-oncology evolves beyond conventional chemotherapy, radiographic timing and response criteria will need to be modified to account for immunotherapeutic interventions. Imaging correlates should follow standardized guidelines while integrating exploratory imaging techniques.

The response assessment in neuro-oncology (RANO) criteria were initially developed to better evaluate imaging response assessment in glioma considering possible radiation- and temozolomide-induced pseudoprogression as well as pseudoresponse following anti-angiogenic therapies such as bevacizumab [26, 27]. A later iteration, the modified RANO (mRANO) criteria, attempted to address limitations by using the post-radiation timepoint as a baseline comparator and eliminating FLAIR sequences; however, this remained agnostic to treatment type [28]. With ACT trials, new challenges have arisen that require further adjustments to previous criteria. Increased lesion size or new enhancing lesions seen radiographically following administration of immunotherapeutics, including ACT, does not exclude therapeutic benefit. Indeed, multiple trials have shown that patients can derive durable clinical benefit despite early pseudoprogression, although the frequency of this phenomenon remains unknown [29]. In response to these findings, and to guide subsequent response criteria, the immunotherapy Response Assessment for Neuro-Oncology (iRANO) criteria were developed [30••]. These criteria allow patients to continue immunotherapy regardless of radiographic findings showing progressive disease by RANO criteria within 6 months from the start of immunotherapy if the patient does not have worsening neurological symptoms definitely related to progressive tumor. As such, progression is confirmed with continued tumor growth on follow-up imaging. The iRANO criteria have not been validated in children yet are commonly incorporated into pediatric ACT trial endpoints due to the lack of alternative guidelines. One of the practical shortcomings of response assessment in CAR T cell trials is de-centralized interpretation of imaging, since most trials for children enroll at a single institution without central eligibility or radiographic review. Using criterion-based guidelines is one way to standardize outcome measures across trials. However, iRANO criteria that rely on the presence or absence of clinical symptoms can be an imprecise differentiator, as profound physical symptoms may result from either tumor progression or on-target inflammatory processes.

Novel imaging technology, using both functional and molecular imaging techniques, may provide clarity in therapeutic response assessment for patients treated with cellular therapies [24, 25, 31, 32]. Examples include advanced MRI, immuno-positron emission tomography (PET), and single-photon emission computed tomography (SPECT). These are being explored as novel imaging studies measuring physiologic or molecular changes that arise from infiltration and/or activation of immune-specific cells and may lead to better prediction of response or progression, allowing patients to remain on therapy if there is possible benefit and to avoid unnecessary toxicity when appropriate. Post-imaging analysis is also advancing quickly, and radiomics can extract high-dimensional imaging features that elude visual review and can inform machine learning models [33–35]. These machine learning algorithms are gaining momentum in accurately predicting clinical outcomes and are now capable of detecting medulloblastoma subgroups, distinguishing posterior fossa tumor subtypes, and prognosticating survival in DIPG [36–38]. Although novel imaging technologies may not be broadly available, relegating them to dedicated centers for the time being, multi-institutional studies could still establish an infrastructure for centralized ACT imaging analysis. Cooperative efforts to standardize imaging interpretation hold promise for improved non-invasive identification and monitoring of ACT responses and immune-related events.

## Chemokine/Cytokine and Immune Effector Cell Analysis

As current neuroimaging is insufficient to assess disease response, and as serial tumor biopsies are frequently ethically and practically challenging, alternative biomarker-based strategies may provide greater insight into therapeutic endpoints and allow real-time clinical decision-making. In particular, correlative assessments of cytokine levels and circulating cells in the CSF and peripheral blood are beginning to reveal key insights into CAR bioactivity and anti-tumor immune responses [19••, 20••, 21••].

The route of CAR T cell administration may be associated with unique cytokine and toxicity profiles. Intravenous (IV) CAR T cell administration for CNS tumors is generally associated with higher proinflammatory cytokine levels in the blood, particularly IL-6, than is observed with intracerebroventricular (ICV) delivery [20••]. In contrast, locoregional infusion of CAR T cells directly into the CNS appears to be associated with higher cytokine levels in the CSF than in the blood, including CCL2, CXCL10, GM-CSF, IL-2, TNF, and IFNɣ [19••, 21••]. Interestingly, CXCL10 (or IP-10), a potent recruiter of effector T cells, appears to be one of the chemokines detected at the highest levels in CSF after locoregional infusion of CAR T cells, across multiple CNS tumors and CAR T cells [19••, 21••]. These early findings suggest that circulating CSF or serum cytokines may be an important biomarker of effector cell function and may even predict treatment success or failure. Therefore, serial chemokine/cytokine collection and analysis should be standardized across trials to allow for a harmonization of results that could guide future trials.

Beyond circulating chemokines/cytokines, paired peripheral blood and CSF sampling also enables an informative evaluation of immune cell populations during therapy. The routine use of flow cytometry permits robust multiparameter characterization of cells recovered from these compartments. Importantly, flow cytometry also allows identification of CAR T cells in these samples, although with less sensitivity than targeted PCR [39]. Multiple studies have demonstrated expansion of CAR T cells in the peripheral blood after IV infusion [10, 20••, 40]. In contrast, IC delivery of CAR T cells has not been associated with robust peripheral blood CAR T expansion except when CAR T cells are delivered shortly after lymphodepletion [19••, 20••, 21••, 39, 41]. However, early findings have shown detectable CAR T cells in the CSF post IC delivery and, in some cases, persistence of circulating CAR T cells over longitudinal CSF collections [21••]. These findings parallel the proinflammatory cytokine data discussed above, underscoring the need for comprehensive multiplex analyses in these trials.

Flow cytometry with single-cell technologies enables highly multiplexed characterization of cell state and identity, including at the level of TCR sequence, transcriptome, and epigenome. Pseudotime and time course analyses permit tracking of clonal evolution and cell fate in the context of the CNS tumor environment, providing deep insight into the anti-tumor immune response (for instance, identifying CAR T cell expansion during times of clinical response). These assays also can provide quantitative analysis of variable immune populations that may be driving CAR T cell efficacy or antagonism. Through multiplexed analyses that are consistent among trials, there is the potential to unlock variability in immune populations based on tumor biology, tumor location, and ACT characteristics, which would otherwise be undetectable in an underpowered single-institution study.

**Fig. 1:**
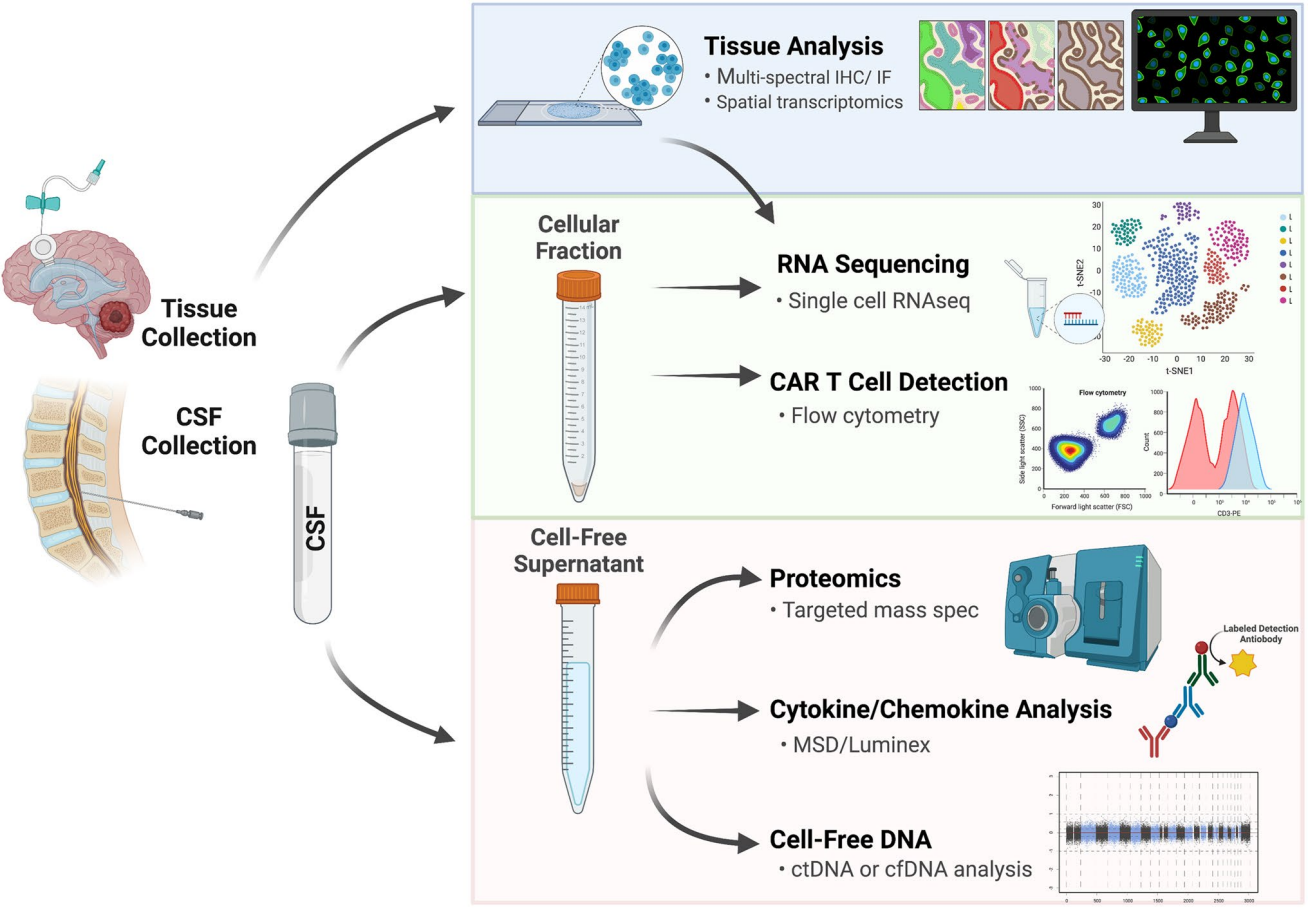
Schematic of correlative study opportunities for biospecimens from pediatric CNS ACT clinical trials (CSF, cerebrospinal fluid; MSD, Meso Scale Discovery platform; ctDNA, circulating tumor DNA; cfDNA, cell-free DNA)

It is important to note that variations in sample collection, handling, flow cytometry panel design, and single-cell techniques can make cross-study comparisons difficult. Standardizing collection, processing, data warehousing, and analysis pipelines will be important for ensuring that future trials are as informative as possible. Technical publications on processing and data alignment, such as the recent report from the CPCI outlining practices in flow cytometry and cytokine/chemokine analysis, are essential in this endeavor [23••]. Figure 1 offers a schematic representation of upstream sample collection and pipelines for data generation in early phase CNS ACT trials.

## Targeted Mass Spectrometry

Monitoring of the immune state will be critically important for improving ACT. This is especially true in CNS tumors, where the Munro-Kellie doctrine imposes a narrow therapeutic window for immune-mediated treatments. Increasingly, it is becoming clear that this monitoring must be both broad and deep if we are to extract meaningful information from these first trials.

Current standard practice typically incorporates multiplex cytokine analysis, which permits the measurement of scores of cytokines (often up to 50). However, there are many biologically active small polypeptides in blood and CSF, and most of them bear important functional post-translational modifications that cannot be detected by conventional analysis. Another developing platform for this purpose is targeted proteomics, which takes advantage of the exquisite sensitivity of liquid chromatography/mass spectrometry (LC–MS/MS) [42, 43]. The Paulovich group has created analyte panels reflective of the immune state, including immune lineage markers (e.g., CD14, CD45) tested upon both tumor tissue and blood plasma [44, 45]. There are many reasons to believe that the proteomically restricted CSF, which is more proximate to CNS tumors, would be very amenable to this type of analysis as well. These panels leverage immuno-multiple reaction monitoring mass spectrometry (immuno-MRM-MS), in which a multiplexed panel of validated antibodies is used to enrich a digested protein lysate for peptides of interest. This platform has been demonstrated to be reproducible in an inter-laboratory validation study [46, 47]. MRM-MS assays require very little analyte, making them potentially suited to clinical applications in which there are limitations in sample quantity (e.g., needle biopsies, CSF, or extracellular fluid). An additional advantage is the ability to assay peptides with post-translational modifications representing protein activation states. The assays have demonstrated remarkable reproducibility, sensitivity, and dynamic range, making them an exciting addition to the translational toolkit [48]. The first assay panel has been CAP/CLIA certified for use by the clinical research community, and there are two additional panels under development.

This approach was piloted in two children with DIPG who received a combined 28 doses of intracerebroventricularly delivered B7-H3 CAR T cells and through analysis of multiple timepoints was able to capture modulation of 50 CSF and 59 serum proteins [21••]. Corresponding with cytokine data, there were fewer protein fluctuations in the serum compared to the CSF, which further supports the importance of CSF-focused correlative assays. While preliminary, several analytes (CD14, CD163, CD44, CSF-1, etc.) tracked between patients, while others, such as the target antigen of the ACT (B7-H3 or CD276) exhibited variability between the two patients [21••]. While analysis in a larger cohort is required, this proof of principle that proteins can be reliability be detected and tracked over time provides a potentially critical avenue to assessing ACT bioactivity, modulation of the local immune microenvironment, and tumor demise or evolution.

## Circulating Genomic Analysis

There have been significant advances in the use of cell-free tumor DNA (cfDNA) in CSF as a “liquid biopsy” technique to detect CNS tumors [49–51]. By identifying fragments of DNA shed from tumor cells, cfDNA assays seek to quantify measurable disease in the CSF and correlate it with overall disease burden, potentially allowing the detection of disease recurrence or progression prior to imaging changes [51, 52]. Methods used for cfDNA detection in CSF include digital droplet PCR, whole exome sequencing, targeted next-generation DNA sequencing for driver mutations, and low-coverage whole genome sequencing (lcWGS) for copy number alterations [50, 52–54]. These methods have variable sensitivity of detection, cost, and processing time but are generally becoming cheaper, faster, and more sensitive. Molecular characterization of the tumor genome following ACT may also assess changes in the tumor mutational profile following surface antigen targeting, both in terms of target alteration (e.g., B7-H3) and cooperating mutations. CSF sampling may also provide a more comprehensive molecular profile than surgical sampling. Using cfDNA to reliably discriminate between treatment changes, pseudoprogression, and true progression would be tremendously beneficial in early phase ACT trials. Ultimately, cfDNA is well-suited for clinical application and could be an additional tool for understanding the biology of ACT.

## Tissue Analysis

Pediatric CNS tumors have been stratified into molecular subtypes through transcriptomics and methylomics [55–57]. While these studies have been informative, most were performed on bulk snap frozen tissue that lack the resolution to fully characterize the cellular heterogeneity of the tumor or its microenvironment (TME). Over the past 5 years, single-cell and single-nuclei RNA-sequencing approaches have advanced our understanding of the diversity of cells in pediatric brain tumors including infiltrating immune cells [58]. These studies provide benchmark datasets to compare cellular subtype changes in the TME following ACT where surgical resection is performed. Single-cell and single-nuclei RNA-sequencing, requiring viably disaggregated cells or snap frozen material respectively, can be applied to ACT correlative studies, as they utilize tissue collected during routine surgical debulking from many primary and recurrent CNS tumors.

Immune cell characterization is feasible through single-cell sequencing techniques but with the caveat that single-cell RNA-sequencing is less feasible due to its requirement for viably disaggregated tumors. Further, immune cell single-cell RNA sequencing requires further validation to determine immune cell function within the tumor/TME. Similar to flow cytometric studies, single-cell RNA-sequencing protocols are not standardized across institutions which can limit the comparison of data between studies conducted in multiple institution.

Additional limitations with single-cell techniques are the loss of spatial orientation that occurs through the dissociation of tissue, as well as inadequate amounts of material to process. When surgical material is available, spatial proteomics can readily be performed on formalin-fixed, paraffin-embedded (FFPE) tissue. Multiplexed immunofluorescent staining, of FFPE samples, using up to 9 antibodies can detect cells within the TME, and subsequent analytics can be applied to the images to (1) measure distances between cell types, (2) quantify the cell subtype, and (3) identify architectural features within the TME and the cell subtypes within the features. In addition, recent work has shown how orchestrating the spatially resolved transcriptomics, and the data from immunohistochemistry, H&E staining, and RNA transcriptomics can be aligned to delineate the cellular subtype, the heterogeneity with the tumor, and the tumor microenvironment with the spatial orientation intact [59]. Utilization of this model is highly encouraged to identify changes within the CNS tumor microenvironment following ACT.

However, unlike CSF and blood collection, where it is feasible to perform serially throughout a trial, serial CNS tissue specimens may not be practical, and there are ethical questions to be asked in serial tumor collections that require neurosurgery and most likely will not change the treatment outcome for that particular child. For studies enrolling patients with recurrent tumors where surgical resection is often performed, it may be possible to give ACT treatments prior to surgery and use historical data as a comparison to changes within the TME. Ultimately, real-time evaluations via this method are impossible with current technologies, and so while direct tissue evaluation is important, timepoints will inherently be limited. Regardless, protocols for tissue collection could still be standardized even in the setting of autopsy, as many patients generously donate tissue at the time of death due to a selfless appreciation for the critical nature of these studies and the potential benefit to future children. Considering the trust that they bravely place into these early phase, and sometimes first-in-human, clinical trials, we have a mandate to maximize our learning from every biospecimen available, especially donations at the end of life.

## Discussion

The growth of early-phase ACT clinical trials in pediatric neurooncology holds great promise for the future and reflects tremendous recent preclinical advances. Many of these trials are small and at single institutions, demanding standardized collaboration across clinical efforts as the most efficient way to improve cancer care for children; this was the motivating principle behind the CPCI. Foremost, we strongly advocate for continued cross-institutional alliances that prioritize scientific discovery and excellence in clinical care while defying barriers in the sharing of biospecimens, data, and advances.

Correlative assessments will be exponentially more valuable if they are collected, processed, and analyzed in a fashion that allows harmonization (Fig. 1). While sample collections vary based on the route of ACT delivery, standard pre- and post-infusion biospecimen collections should be prioritized, especially from the CSF. Other significant timepoints should include periods of potential pseudoprogression/progression or acute toxicity, providing critical comparators for safety and efficacy. In the setting of intracranial ACT administration, peripheral collections may be less instructive so it is reasonable to limit their processing after initial evaluations are reassuring for efficiency. For patients receiving intracranial ACT, serial CSF collections likely can be limited to CNS access devices, such as Ommaya catheters or ventriculoperitoneal shunts for procedural practicality, limiting anatomical variability and avoiding anesthesia exposure that accompanies lumbar puncture procedures in the pediatric setting [60••]. Standardization of assessments, including cytokines, proteomics, and cfDNA, may offer a future path toward triangulating clinical changes to initially allow better interpretation of clinical data at study completion, but considering the speed of technological advancement may ultimately be used for real-time assessments. These combined metrics could guide when to continue or abandon a therapy, as well as how to sequence therapy in a truly personalized approach.

For radiographic endpoints, pediatric ACT clinical trials currently may be best served to incorporate iRANO criteria with a goal of centralized review within immunotherapy consortiums. Early adoption of machine learning and advanced imaging techniques may be critical, enabling the obsolescence of imaging sequences that are less helpful for these patients. Ultimately, considering the lack of validation of iRANO for children and the inherent complexity of determinant progression-free survival due to pseudoprogressive symptoms, we advocate that overall survival be the central response and survival outcome for pediatric CNS ACT trials. Hopefully a future state will allow incorporation of other prospectively validating imaging and correlative modalities based on the frameworks we and others aim to provide. This near future of even more sophisticated pediatric CNS ACT trials with broadened correlative science objectives will demand data warehousing and infrastructure to become even more vital. Initiatives like the Children’s Brain Tumor Network (CBTN), a partnership among international researchers to collect the rich supply of molecular, imaging, and clinical data available through consortium-led trials, will allow the ACT field to better share and interpret collective data.

## Conclusion

The field of ACT is advancing rapidly, particularly for children with CNS tumors. As initial pioneering trials reach the first clinical crossroads, we should embrace the opportunity to collaborate rather than compete. Current early phase trials offer hope and inherently strive for therapeutic success, but these studies also importantly serve as the seed for enhanced future studies. To this aim, a critical step in the maturation of our field will be developing standards for the systematic collection, analysis, storage, and understanding of serialized correlative assessments of, in particular, CSF biomarkers. With parallel progress surely coming in genomics and neuroimaging, we have the ability to collect patient biospecimens now to maximize the learning from this first generation of pediatric CNS ACT clinical trials. Through technological progress, and most importantly broad cooperation, there are innumerable opportunities to optimize future trials and ultimately reach the most significant goal: curing the currently incurable tumors of childhood.
